# Association of Perfluoroalkyl and Polyfluoroalkyl Substances With Adiposity

**DOI:** 10.1001/jamanetworkopen.2018.1493

**Published:** 2018-08-31

**Authors:** Andres Cardenas, Russ Hauser, Diane R. Gold, Ken P. Kleinman, Marie-France Hivert, Abby F. Fleisch, Pi-I D. Lin, Antonia M. Calafat, Thomas F. Webster, Edward S. Horton, Emily Oken

**Affiliations:** 1 Division of Chronic Disease Research Across the Lifecourse, Department of Population Medicine, Harvard Medical School, Harvard Pilgrim Health Care Institute, Boston, Massachusetts; 2 Department of Environmental Health, Harvard T.H. Chan School of Public Health, Boston, Massachusetts; 3 Channing Division of Network Medicine, Brigham and Women’s Hospital, Boston, Massachusetts; 4 Department of Biostatistics and Epidemiology, University of Massachusetts–Amherst School of Public Health and Health Sciences, Amherst; 5 Diabetes Unit, Massachusetts General Hospital, Boston; 6 Division of Pediatric Endocrinology and Diabetes, Maine Medical Center, Portland; 7 Center for Outcomes Research and Evaluation, Maine Medical Center Research Institute, Portland; 8 Division of Laboratory Sciences, National Center for Environmental Health, Centers for Disease Control and Prevention, Atlanta, Georgia; 9 Department of Environmental Health, Boston University School of Public Health, Boston, Massachusetts; 10 Joslin Diabetes Center, Harvard Medical School, Boston, Massachusetts

## Abstract

**Importance:**

Perfluoroalkyl and polyfluoroalkyl substances (PFASs) are ubiquitous synthetic chemicals that are suspected endocrine disruptors.

**Objectives:**

To determine the extent to which PFASs are associated with increases in weight and body size and evaluate whether a lifestyle intervention modifies this association.

**Design, Setting, and Participants:**

This prospective cohort study included 957 individuals who participated in the Diabetes Prevention Program trial, conducted from July 1996 to May 2001, and the Diabetes Prevention Program Outcomes Study, conducted from September 2002 to January 2014. Statistical analysis was conducted from September 1, 2017, to May 25, 2018.

**Interventions and Exposures:**

The initial lifestyle intervention consisted of training in diet, physical activity, and behavior modification, with the major goals of achieving 7% weight loss with subsequent maintenance and a minimum of 150 minutes per week of physical activity. Participants randomized to placebo received standard information about diet and exercise. A total of 6 plasma PFASs were quantified at baseline and 2 years after randomization, means were calculated from baseline and year 2 concentrations, and means were summed to assess total PFAS burden.

**Main Outcomes and Measures:**

Weight, waist circumference, and hip girth were measured at baseline and at scheduled visits.

**Results:**

Of the 957 participants, 625 (65.3%) were women and 731 participants (76.4%) were between 40 and 64 years of age; 481 participants were randomized to the lifestyle intervention and 476 participants were randomized to the placebo arm. The PFAS concentrations were not different by treatment arm and were similar to concentrations reported for the US population in 1999-2000. The association of PFAS and weight change differed by treatment. Each doubling in total PFAS concentration was associated with an increase of 1.80 kg (95% CI, 0.43-3.17 kg; *P* = .01) from baseline to 9 years after randomization for the placebo group but not the lifestyle intervention group (−0.59 kg; 95% CI, –1.80 to 0.62 kg; *P* = .34). Similarly, each doubling in PFAS was associated with a 1.03-cm increase in hip girth in the Diabetes Prevention Program trial for the placebo group (95% CI, 0.18-1.88 cm; *P* = .02) but not the lifestyle intervention group (−0.09 cm; 95% CI, −0.82 to 0.63 cm; *P* = .80). No associations were observed for changes in mean waist circumference.

**Conclusions and Relevance:**

Among adults at high risk for diabetes, higher plasma PFAS concentration was associated with increases in weight and hip girth over time, but a lifestyle intervention attenuated these associations. Diet and exercise may mitigate the obesogenic effects of environmental chemicals.

**Trial Registration:**

ClinicalTrials.gov Identifier: NCT00004992 and NCT00038727

## Key Points

**Question:**

Are synthetic perfluoroalkyl and polyfluoroalkyl substances associated with changes in weight, body size, and adiposity in adults?

**Findings:**

In this follow-up cohort study of 957 participants in the the Diabetes Prevention Program Outcomes Study for 15 years, plasma concentrations of perfluoroalkyl and polyfluoroalkyl substances were associated with increases in weight gain and body size among individuals initially randomized to placebo. However, for participants randomized to a lifestyle intervention of exercise and diet, no detrimental associations were observed.

**Meaning:**

Plasma concentration of perfluoroalkyl and polyfluoroalkyl substances is associated with increases in weight and body size in adults; however, exercise and diet may modify and attenuate the obesogenic effects of perfluoroalkyl and polyfluoroalkyl substances.

## Teaser

This cohort study from the Diabetes Prevention Program trial and Outcomes Study determines the extent to which perfluoroalkyl and polyfluoroalkyl substances are associated with increases in body weight and size and evaluates whether a lifestyle intervention modifies this association.

## Introduction

Perfluoroalkyl and polyfluoroalkyl substances (PFASs) are a group of synthetic chemicals that persist in humans and the ecosystem and are universally detected across populations worldwide.^1,2,3,4,5,6^ Nearly the entire US general population (>95%) has detectable serum concentrations of several PFASs.^7^ Many PFASs have strong chemical and thermal stability, with both hydrophobic and hydrophilic groups, that make them valuable for industrial applications but resistant to environmental and physiological degradation.^8,9^ Perfluoroalkyl and polyfluoroalkyl substances have been used in nonstick cookware, oil- and water-resistant textiles, greaseproof food packaging, personal care products, floor polish, and firefighting foams and as industrial surfactants.^10^ Human exposure to PFASs occurs through direct and indirect sources including contaminated drinking water, food, personal care products, soil, dust, and air.^11,12^

Perfluoroalkyl and polyfluoroalkyl substances bind to blood proteins, and many bioaccumulate in the body, with half-lives ranging from 3.5 to 8.5 years.^13^ Some PFASs can change membrane fluidity and signaling for cell receptors,^14^ and have endocrine-disrupting effects in vitro.^15,16^ The activation of multiple nuclear receptors by PFASs,^17^ including the peroxisome proliferator–activated receptor α,^18^ is hypothesized to alter metabolic regulation, documented in animal studies.^19^ In addition, thyroid hormone dysregulation and changes in resting metabolic rate are associated with PFAS exposure, potentially leading to weight gain.^20^ However, several mostly cross-sectional epidemiologic studies of associations for PFASs with weight, body size, or adiposity have reported mixed findings, including both positive and inverse effects.^21,22,23,24^

We measured plasma PFAS concentrations at baseline and during the second annual visit of the Diabetes Prevention Program (DPP) trial that has followed participants prospectively for approximately 15 years after randomization as part of the Diabetes Prevention Program Outcomes Study (DPPOS) cohort. We hypothesized that higher plasma concentrations of PFASs would be associated with greater increases in weight and body size during follow-up. Moreover, we postulated that the lifestyle intervention, consisting of diet and exercise, would modify this association, attenuating the obesogenic effects of PFASs compared with placebo.

## Methods

### Study Population

The DPP, a multicenter randomized clinical trial to prevent or delay type 2 diabetes among high-risk individuals with elevated fasting plasma glucose levels, recruited participants from 27 clinical centers across the United States between July 1996 and May 1999. Inclusion criteria were age 25 years or older, body mass index (BMI) (calculated as weight in kilograms divided by height in meters squared) of 24 or greater (≥22 among Asian Americans), and fasting glucose concentration of 95 to 125 mg/dL and 140 to 199 mg/dL measured 2 hours after a 75-g oral glucose load (to convert glucose to millimoles per liter, multiply by 0.0555).^25^ The DPP aimed to recruit at least half the participants to be women and approximately half the participants from ethnic minorities (African American, Hispanic, American Indian, Asian American, and Pacific Islander).^25^ Participants were randomized to a pharmacologic intervention (metformin), a placebo-treated control, or a lifestyle intervention group receiving intensive training in diet, physical activity, and behavior modification.^26^ The lifestyle intervention had 2 major goals: achieving a 7% weight loss with subsequent maintenance, and a minimum of 150 minutes per week of physical activity similar in intensity to brisk walking.^26^ Dietary changes were initially focused on reducing fat intake and then introducing the maintenance of caloric balance to lose 0.45 to 0.91 kg per week.^27^ Participants assigned to the placebo group received standard information about diet and exercise but no motivational counseling. The original aims, design, and baseline characteristics of the DPP have been previously reported.^25,28^ The DPP was terminated in May 2001, based on the effectiveness of both the lifestyle and pharmacologic interventions for preventing type 2 diabetes.^26^ Masked treatment was discontinued in July 2001 and, during a 13-month bridge period between the DPP and DPPOS protocols, all participants were offered a modified version of the lifestyle intervention.^29,30^ Study participants have been followed up in DPPOS (September 2002 to January 2014) for approximately 15 years.^31^ The institutional review board at each clinical center approved the protocol, and all participants provided written informed consent for DPP and DPPOS.^26^ For the present study, data were deidentified and the Harvard Pilgrim Health Care institutional review board reviewed and approved all study protocols. The involvement of the Centers for Disease Control and Prevention laboratory did not constitute engagement in human subjects research. This study followed the Strengthening the Reporting of Observational Studies in Epidemiology (STROBE) guidelines.^32^

For the current study, we selected individuals in the lifestyle intervention and placebo arms of the trial with available stored plasma samples collected at baseline and during the second year of the DPP. No samples from the pharmacologic arm of the trial were quantified for PFASs. We did not include participants from the pharmacologic intervention (metformin) because of unknown interactions or kinetics between PFASs and the pharmacologic intervention. A total of 957 participants were eligible for quantification of plasma PFAS concentrations in the placebo and lifestyle intervention groups.

### Plasma PFAS Concentrations

Blood plasma samples collected during the DPP were subsequently stored at the National Institute of Diabetes and Digestive and Kidney Diseases repository (https://repository.niddk.nih.gov/home), and later shipped from the repository to the Centers for Disease Control and Prevention for analyses. We quantified 6 distinct PFASs using a modification of an online solid-phase extraction–high-performance liquid chromatography–isotope dilution–tandem mass spectrometry approach described previously^33,34^; the limit of detection was 0.1 ng/mL for all analytes (eTable 1 in the Supplement). All 6 PFASs—perfluorooctane sulfonic acid (PFOS), perfluorooctanoic acid (PFOA), perfluorohexane sulfonic acid, *N*-ethyl-perfluorooctane sulfonamido acetic acid, *N*-methyl-perfluorooctane sulfonamido acetic acid (Me-PFOSA-AcOH), and perfluorononanoic acid (PFNA)—were detected in more than 80% of the samples; concentrations below the limit of detection were replaced by the limit of detection/√2.^35^ We summed all 6 PFAS concentrations in nanograms per milliliter at baseline to estimate total baseline PFAS burden to be used in baseline cross-sectional analyses. In addition, because of the long half-life and nonsignificant changes in concentrations during the first 2 years of the DPP, we calculated means of baseline and year 2 concentrations for each of the 6 PFASs and summed all 6 means to better reflect total mean PFAS body burden for models that examined long-term changes from baseline in weight, waist circumference, and hip girth.

### Anthropometric Measurements

Weight was measured at baseline and semiannually thereafter during the DPP and DPPOS. At each study visit, trained and certified research staff performed all measures according to protocols.^36^ Weight was measured twice on a calibrated balance scale to the nearest 0.1 kg and a third measurement was taken if the first 2 measurements were not within 0.2 kg.^36^ We calculated mean participant weight in kilograms at each visit. Waist circumference was measured annually in the DPP and DPPOS twice in centimeters and a third measure was taken if the first 2 measurements were not within 0.5 cm. Waist was defined as the midpoint between the highest point of the iliac crest and the lowest part of the costal margin in the midaxillary line. Hip girth was measured at baseline and annually thereafter during the DPP but not in DPPOS, at the level of the greater femoral trochanters.^37^ Subscapular, triceps, suprailiac, abdominal, and medial calf skinfold thicknesses were measured in millimeters 3 times at baseline using calibrated Lange skinfold calipers.^38,39^ We calculated the mean for the 3 measurements for the 5 body skinfolds and then summed all 5 mean measurements to calculate total body skinfold thickness. Finally, we subtracted baseline measurements for each participant from the measured weight, waist circumference, and hip girth at each visit to obtain the change from baseline. Visceral fat and subcutaneous fat were measured at baseline between the L2-L3 and L4-L5 vertebrae using computed tomography among a random subsample of 348 participants.

### Covariates

We obtained deidentified participant data from the National Institute of Diabetes and Digestive and Kidney Diseases repository and adjusted all multivariate models for the following baseline demographic characteristics selected a priori: sex, self-reported race/ethnicity, age, height, educational level, smoking history, marital status, and annual household income. Time in days from baseline for each outcome assessment was used in longitudinal models with repeated measurements.

### Statistical Analysis

Statistical analysis was conducted from September 1, 2017, to May 25, 2018. We calculated geometric means and interquartile ranges for PFAS concentrations for both total baseline and mean PFAS across demographic characteristics. We used a Wilcoxon rank-sum test or a Kruskal-Wallis test to examine unadjusted concentration differences across participants’ baseline characteristics. We compared PFAS concentrations in our study with reported geometric means of selected PFAS concentrations for the US population (National Health and Nutrition Examination Survey, 1999-2014) using bar plots.^40^ We calculated Spearman correlation coefficients (*r*) among mean PFAS concentrations. Distributions of total baseline and mean PFAS concentrations were right skewed and thus we log_2_-transformed them for multivariate analyses. We used adjusted linear regression models to estimate cross-sectional associations of total baseline PFAS concentrations with weight, waist circumference, hip girth, sum of skinfold thicknesses, and subcutaneous and visceral fat area.

In longitudinal models we tested for effect modification by baseline treatment with multiplicative interactions of baseline treatment assignment and total log_2_-transformed mean PFASs (see model equations in the eAppendix in the Supplement). To test for prospective associations during follow-up, we used longitudinal mixed-effects regression models with random intercepts and slopes to estimate associations of total log_2_-transformed mean PFAS concentrations and changes in weight, waist circumference, and hip girth stratified by treatment assignment. Sensitivity analyses were performed using total baseline PFAS concentrations. We used the *lme4* package of R statistical software (R Foundation for Statistical Computing) to fit mixed-effects regression models.^41^ Visual inspection of scatterplots and means for physical measurements over time suggested nonlinear trajectories for changes in weight and waist circumference (eFigure 1 in the Supplement). Therefore, we also included the fixed effects of follow-up time squared as well as the interaction between follow-up time squared and total mean PFAS concentrations for changes in weight and waist circumference. The change in hip girth from baseline was linear. Using fully adjusted models we plotted estimated trajectories of changes in weight, waist circumference, and hip girth by the 25th and 75th percentiles of total mean PFAS concentrations. We plotted longitudinal model trajectories holding categorical variables at their most frequent level and height fixed at the median while varying PFAS. Although the shape of the trajectories will be different at different covariate levels, the association with PFAS will be constant. Longitudinal regression model equations used for each outcome are outlined in the eAppendix in the Supplement.

To aid in the interpretation of our longitudinal models we estimated changes in weight and waist circumference by total mean PFAS concentrations at the fifth annual DPPOS visit, which occurred approximately 9 years after randomization, using adjusted linear regression models for weight and waist circumference change from baseline. We calculated point estimates at the fifth annual DPPOS visit because we had outcome information for more than 80% of the initial participants (eTable 2 in the Supplement). All *P* values were from 2-sided tests and results were deemed statistically significant at *P* < .05. Data management and analyses were performed in R, version 3.4.1 (The R Foundation for Statistical Computing).

## Results

### Participant Characteristics and Plasma PFASs

We included a total of 957 participants (625 women, 332 men; 731 [76.4%] between 40 and 64 years of age): 481 participants from the lifestyle intervention group of the DPP and 476 participants from the placebo group with available stored plasma samples. Concentrations of total baseline and mean PFASs differed by sex, race/ethnicity, and educational level. Total concentration of mean but not baseline PFASs differed by baseline household income and were similar across age, body mass index classification, smoking history, and marital status. As expected, geometric means for total baseline and mean PFAS concentrations were not significantly different in the 2 arms of the trial (Table 1). Baseline characteristics were not different between the 2 arms of the trial. Characteristics of participants included in this study were similar to those in the overall DPP study.^34^

**Table 1.** Demographic Characteristics and Distribution of Baseline and Mean Baseline and Year 2 Sum PFAS Concentrations in the Diabetes Prevention Program

Characteristic	Participants, No. (%)	Total Baseline PFASs, ng/mL		Total Mean PFASs, ng/mL	
		GM (IQR)	*P* Value	GM (IQR)	*P* Value
Overall, No.	957	38.0 (28.6)		40.6 (25.7)	
Sex					
Male	332 (34.7)	41.9 (29.3)	<.001	44.8 (25.1)	<.001
Female	625 (65.3)	36.7 (28.6)		38.5 (25.5)	
Race/ethnicity					
Non-Hispanic white	552 (57.7)	38.3 (26.9)	<.001	40.7 (24.7)	<.001
African American	184 (19.2)	43.8 (36.4)		46.5 (33.7)	
Hispanic of any race	179 (18.7)	33.6 (27.9)		34.9 (25.3)	
All other	42 (4.4)	40.3 (33.0)		40.3 (22.0)	
Age, y					
<40	112 (11.7)	39.1 (28.8)	.35	40.8 (22.7)	.16
40-44	107 (11.2)	35.6 (27.3)		36.4 (21.7)	
45-49	213 (22.3)	37.6 (32.1)		39.7 (27.0)	
50-54	167 (17.5)	40.9 (28.4)		42.4 (30.2)	
55-59	137 (14.3)	41.0 (29.3)		43.4 (27.2)	
60-64	107 (11.2)	35.9 (29.3)		39.3 (26.8)	
≥65	114 (11.9)	38.0 (24.3)		41.4 (22.4)	
BMI classification^a^					
Normal (18.5-24.9)	22 (2.3)	31.2 (31.3)	.25	34.5 (22.2)	.43
Overweight (25.0-29.9)	287 (30.0)	37.8 (28.9)		40.1 (25.9)	
Obese (>30.0)	648 (67.7)	39.0 (28.4)		41.0 (25.3)	
Educational level					
Less than high school	45 (4.7)	26.5 (25.4)	<.001	26.9 (20.4)	<.001
High school or GED	200 (20.9)	41.6 (29.9)		42.7 (28.3)	
College	469 (49.0)	39.1 (30.3)		41.2 (26.3)	
Graduate school	243 (25.4)	37.4 (26.2)		40.6 (24.2)	
Smoking history					
Nonsmoker	544 (56.8)	37.9 (28.8)	.37	39.6 (25.7)	.12
Former	356 (37.2)	38.9 (28.4)		41.5 (24.7)	
Current	57 (6.0)	41.1 (33.2)		43.7 (28.7)	
Marital status					
Married or cohabitating	647 (67.6)	38.8 (27.6)	.29	40.8 (25.8)	.18
Single	114 (11.9)	40.0 (30.1)		42.5 (23.1)	
Divorced or separated	152 (15.9)	35.4 (27.4)		37.9 (23.4)	
Widowed	44 (4.6)	39.9 (46.2)		42.4 (31.0)	
Treatment group					
Lifestyle intervention	481 (50.3)	39.6 (30.1)	.08	41.5 (25.3)	.14
Placebo	476 (49.7)	37.3 (27.4)		39.6 (25.4)	
Annual household income, $					
<20 000	121 (12.6)	33.6 (18.0)	.14	34.9 (25.8)	.02
20 000 to <35 000	167 (17.5)	37.3 (17.9)		39.7 (24.6)	
35 000 to <50 000	194 (20.3)	40.2 (17.3)		41.7 (23.7)	
50 000 to <75 000	184 (19.2)	39.5 (20.9)		42.2 (27.4)	
>75 000	214 (22.4)	40.1 (21.9)		42.6 (25.5)	
Refused to answer	77 (8.0)	38.0 (17.9)		39.9 (22.3)	

Abbreviations: BMI, body mass index; GED, General Educational Development; GM, geometric mean; IQR, interquartile range; PFASs, perfluoroalkyl and polyfluoroalkyl substances.

^a^ Calculated as weight in kilograms divided by height in meters squared.

**Figure 1.**
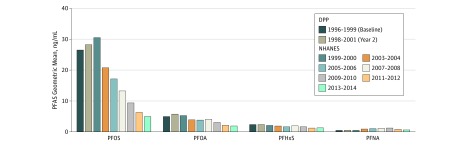
Geometric Means for Select Perfluoroalkyl and Polyfluoroalkyl Substances (PFASs) Measured in the US National Health and Nutrition Examination Survey (NHANES) 1999-2014 and the Diabetes Prevention Program (DPP) at Baseline and Year 2 Trends in PFASs for the US population as well as the study sample. PFHxS indicates perfluorohexane sulfonic acid; PFNA, perfluorononanoic acid; PFOA, perfluorooctanoic acid; and PFOS, perfluorooctane sulfonic acid.

The mean concentrations for the 6 unique plasma PFASs were positively and significantly correlated with one another (range of *r*_S_ across compounds, 0.08-0.61; eFigure 2 in the Supplement). Geometric means of selected plasma PFAS concentrations measured at baseline (1996-1999) and during year 2 of the DPP (1998-2001) were similar to concentrations measured in the US population between 1999 and 2000 but were higher compared with concentrations reported between 2013 and 2014, except for PFNA, which was similar to recent US population measurements (Figure 1).^40^

### Cross-sectional Associations of PFAS Concentrations With Anthropometric Measurements

**Figure 2.**
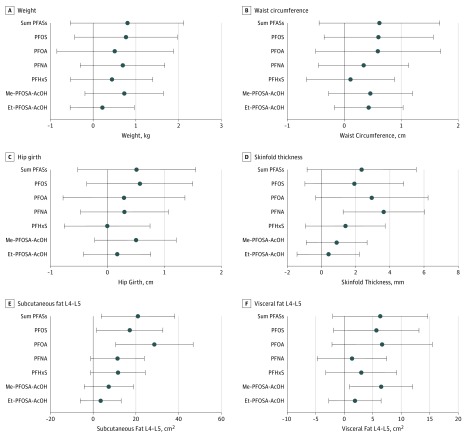
Estimates and 95% CIs Among Adjusted Cross-sectional Associations of Outcomes per Doubling in Perfluoroalkyl and Polyfluoroalkyl Substance (PFAS) Concentrations Measured at Baseline Cross-sectional associations of PFASs and adiposity. Et-PFOSA-AcOH indicates *N*-ethyl-perfluorooctane sulfonamido acetic acid; Me-PFOSA-AcOH, *N*-methyl-perfluorooctane sulfonamido acetic acid; L4-L5, lumbar vertebrae; PFHxS, perfluorohexane sulfonic acid; PFNA, perfluorononanoic acid; PFOA, perfluorooctanoic acid; and PFOS, perfluorooctane sulfonic acid.

In fully adjusted cross-sectional analyses, total baseline PFAS concentrations and individual compounds were not significantly associated with weight, waist circumference, or hip girth. However, a doubling in PFNA was associated with 3.65-mm greater baseline skinfold thickness (95% CI, 1.27-6.02; *P* = .003). Subcutaneous fat area measured by computed tomography between the L4-L5 vertebrae at baseline was directly associated with baseline PFAS (21 cm^2^ greater per doubling in exposure; 95% CI, 3.78-38.26 cm^2^; *P* = .02), PFOS (17 cm^2^ per doubling; 95% CI, 1.61-32.65 cm^2^; *P* = .03), and PFOA (29 cm^2^ per doubling; 95% CI, 10.60-46.74 cm^2^; *P* = .001) concentrations. A doubling in Me-PFOSA-AcOH was associated with a 6.47-cm^2^ greater visceral fat area between the L4-L5 vertebrae (95% CI, 0.95-11.99 cm^2^; *P* = .02). Results for the L2-L3 fat area measurements were similar. Adjusted cross-sectional associations are summarized in Figure 2.

### PFAS Concentrations and Changes in Anthropometric Measurements in DPP and DPPOS

Most participants were followed up for 15 years on average after initial randomization during scheduled semiannual or annual visits in the DPP and DPPOS (eTable 2 in the Supplement). As previously reported for the entire DPP and DPPOS cohort, we also observed the largest decrease in weight, waist circumference, and hip girth 6 months to 1 year after randomization for the lifestyle intervention group, followed by a gradual rebound thereafter. Weight and waist circumference measurements in the placebo group decreased slightly after the introduction of the lifestyle intervention during the bridge period between the DPP and DPPOS (eFigure 1 in the Supplement).^29,31^

While significant interactions were present between PFAS concentrations and baseline treatment assignment in longitudinal models of change in weight and waist circumference, they were not significant for change in hip girth. Therefore, we stratified all longitudinal models by baseline treatment assignment.

**Figure 3.**
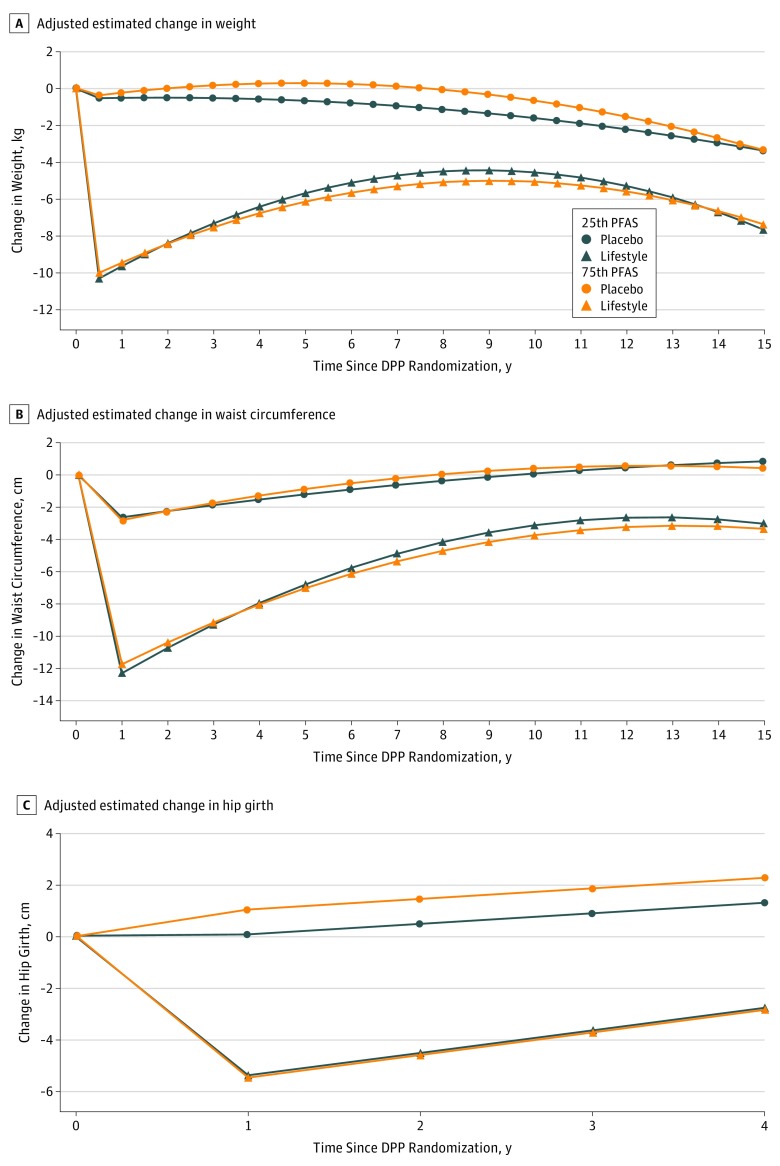
Adjusted Estimated Change in Weight and Body Size From Baseline at the 25th and 75th Percentiles of Total Mean Perfluoroalkyl and Polyfluoroalkyl Substances (PFASs) A, Adjusted estimated change in weight from baseline at the 25th and 75th percentiles of total mean PFASs. B, Adjusted estimated change in waist circumference from baseline at the 25th and 75th percentiles of total mean PFASs. C, Adjusted estimated change in hip girth from baseline at the 25th and 75th percentiles of total mean PFASs. DPP indicates Diabetes Prevention Program.

In longitudinal models, mean PFAS plasma concentration was associated with higher nonlinear weight trajectories for the placebo group but lower weight trajectories for the lifestyle intervention group. Figure 3A shows modeled change by study group in weights at constant values of adjustment covariates at each follow-up visit for those at the 25th and 75th percentiles of mean PFAS concentrations. To estimate the magnitude of this difference, we also calculated change in weight from baseline measured at the fifth annual DPPOS visit (approximately 9 years after randomization) by total mean PFAS concentration. At this time point, each doubling in total mean PFAS plasma concentration was associated with an increase of 1.80 kg (95% CI, 0.43-3.17 kg; *P* = .01) for the placebo group, but no significant weight change for the lifestyle intervention group (−0.59 kg; 95% CI, –1.80 to 0.62 kg; *P* = .34), consistent with longitudinal model trajectories. Among individual PFAS compounds, the mean of baseline and year 2 concentrations of PFOS, Me-PFOSA-AcOH, and PFNA were significantly associated with an increase in weight for the placebo group approximately 9 years after randomization. However, for the lifestyle intervention group, associations were relatively small, not significant, and in the opposite direction, consistent with findings from longitudinal models (Table 2). These associations remain consistent when using only the total of baseline PFAS concentrations or the total of individual baseline analytes (eTable 3 in the Supplement).

**Table 2.** Estimated Adjusted Difference in Mean Weight Change From Baseline to Fifth Annual Diabetes Prevention Program Outcomes Study Visit, per Doubling in Mean Plasma PFAS Concentration and Stratified by Initial Treatment Assignment^a^^,^^b^

PFASs^c^	Lifestyle Intervention Group (n = 402)		Placebo Group (n = 400)	
	Weight Change From Baseline per Doubling in PFASs (95% CI), kg	*P* Value	Weight Change From Baseline per Doubling in PFASs (95% CI), kg	*P* Value
Total mean PFASs	−0.59 (−1.80 to 0.62)	.34	1.80 (0.43 to 3.17)	.01
PFOS	−0.65 (−1.79 to 0.48)	.26	1.70 (0.44 to 2.95)	.008
PFOA	−0.03 (−1.19 to 1.13)	.96	0.92 (−0.41 to 2.25)	.17
PFHxS	−0.08 (−0.90 to 0.73)	.84	0.07 (−0.93 to 1.07)	.89
Et-PFOSA-AcOH	−0.03 (−0.70 to 0.63)	.92	0.68 (−0.13 to 1.50)	.10
Me-PFOSA-AcOH	0.05 (−0.84 to 0.94)	.91	1.33 (0.28 to 2.39)	.01
PFNA	−0.11 (−1.07 to 0.85)	.82	1.06 (0.01 to 2.11)	.04

Abbreviations: Et-PFOSA-AcOH, *N*-ethyl-perfluorooctane sulfonamido acetic acid; Me-PFOSA-AcOH, *N*-methyl-perfluorooctane sulfonamido acetic acid; PFASs, perfluoroalkyl and polyfluoroalkyl substances; PFHxS, perfluorohexane sulfonic acid; PFNA, perfluorononanoic acid; PFOA, perfluorooctanoic acid; PFOS, perfluorooctane sulfonic acid.

^a^ Mean of 9 years of follow-up after randomization.

^b^ Adjusted for participant sex, race/ethnicity, height, age (categorical), marital status (categorical), educational level (categorical), income (categorical), and smoking history (categorical).

^c^ Mean of baseline and year 2 plasma concentrations.

Estimated trajectories for the change in mean waist circumference indicated that higher total mean PFAS concentration was associated with a small gain in waist circumference in the placebo group, but less gain in waist circumference for the lifestyle intervention group, compared with lower PFAS exposure. Figure 3B shows estimated trajectories for the 25th and 75th percentile of total mean PFAS concentration. However, when examining point estimates 9 years after randomization, these changes in waist circumference were relatively small and not statistically significant. For example, at the fifth annual DPPOS visit, the total mean PFAS concentration was not associated with waist circumference for the placebo (0.34 cm; 95% CI, −0.99 to 1.68 cm; *P* = .61) or the lifestyle intervention group (−0.39 cm; 95% CI, −1.54 to 0.76 cm; *P* = .50) (eTable 4 in the Supplement). Finally, each doubling in total mean PFAS concentration was associated with a constant linear increase of 1.03 cm in hip girth (95% CI, 0.18-1.88 cm; *P* = .02) for the placebo group during DPP follow-up. No association was observed in the lifestyle intervention group (−0.09 cm; 95% CI, −0.82 to 0.63 cm; *P* = .80) (Figure 3C). Sensitivity analyses also tested prospective associations using total baseline PFAS concentrations and observed similar results (eFigure 3 in the Supplement).

## Discussion

In this study of overweight and obese adults at high risk of type 2 diabetes randomized to a lifestyle intervention or placebo, we observed that some plasma PFAS concentrations were cross-sectionally associated with higher summed skinfold thicknesses and subcutaneous and visceral fat but not weight, waist circumference, or hip girth prior to randomization. However, during follow-up we observed that greater plasma PFAS concentrations were associated with an increase in weight trajectories and hip girth for the placebo group but not the lifestyle intervention group. This finding may suggest that PFASs act as obesogens only in the presence of other risk factors for obesity, but not when these risk factors are reduced. Overall, these results support our hypothesis that lifestyle changes of exercise and diet can attenuate the obesogenic effects of environmental exposures.

At baseline, we observed associations to be in the hypothesized direction, with baseline PFAS concentrations positively associated with weight, waist circumference, hip girth, skinfold thicknesses, and subcutaneous and visceral fat, but most associations did not reach statistical significance and we did not adjust for multiple comparisons. Some of the cross-sectional association might be due to chance and should be interpreted with caution. Consistent with our prospective positive findings but mostly null cross-sectional associations, the European Youth Heart Study observed almost no evidence of cross-sectional associations of PFOS or PFOA with body size measurements at age 8 to 10 years^42^ or in adolescence.^23^ However, childhood PFOS and PFOA concentrations were directly associated with adolescent (approximately 16 years of age) body mass index, waist circumference, and skinfolds.^23^ In the US population, using biomonitoring measurements from the National Health and Nutrition Examination Survey, cross-sectional associations have been inconsistent for PFAS concentrations, body size measurements, or markers of metabolic syndrome^21,24,43^; these inconsistencies were also seen in the Canadian Health Measures Survey.^44^

The hypothesis of environmental chemicals acting as obesogens not by directly causing obesity but, rather, by modifying sensitivity to other risk factors for obesity was initially proposed by Grün and Blumberg.^45^ There is a dearth of data, both epidemiologic or experimental, on whether PFASs in the presence of other risk factors for adiposity act like obesogens and whether reduction of these other risk factors through interventions may reduce the obesogenic effects of PFASs. However, one double-blind, randomized, placebo-controlled crossover trial of Japanese adults reported that PFOS and perfluorododecanoic acid were associated with homeostatic model assessment of insulin resistance and oxidative stress biomarkers at baseline, but a 4-week supplementation of vitamin C reduced these associations.^46^ It is also possible that increased physical activity, known to favorably modify the balance of antioxidants and prooxidants,^47^ could have played a role. For example, in a recent trial of diet-induced weight loss, baseline plasma PFAS concentrations were associated with greater weight regain, consistent with our results for the placebo group.^20^ In that trial participants initially lost weight on 4 different diets, yet obesogenic associations with PFASs remained. This finding suggests that increased physical activity in our study might have played a role in attenuating obesogenic associations for the lifestyle intervention group.

The observed paradoxical association in the lifestyle group of very small but nonsignificant decreases in weight and waist circumference associated with PFASs could result from confounding. For example, such confounding might have occurred if more physically active participants in the lifestyle intervention group were most exposed to PFASs through sports and outdoor garments, previously reported to be a source of exposure.^48,49^ This hypothesis was postulated by the European Youth Heart Study, in which investigators observed lower waist circumference with higher PFOA concentrations cross-sectionally.^23^ In addition, diet is a major source of PFASs and fast food might be an important route of exposure,^50,51^ as might fish consumption,^52,53^ potentially confounding associations bidirectionally. However, we measured plasma PFAS concentrations at baseline, measured change in body outcomes prospectively, and participants were randomized to study arms, minimizing but not eliminating the chance for dietary habits to confound the results. In addition, at the end of the DPP trial, participants were offered a modified version of the lifestyle intervention. Uptake and adherence to this modified version of the initial intervention could have also hampered the initial associations. Nevertheless, obesogenic associations in the placebo group persisted up to 9 years after randomization.

### Limitations and Strengths

Important limitations of our study include the nongeneralizable results, given that all participants were overweight or obese and had glycemic levels in prediabetes ranges. In addition, participants were randomized to an intensive lifestyle intervention or placebo-treated group, which could limit the generalizability of the study. Concentrations of some PFASs measured in our study have declined for the US population; therefore, concentrations in our study may be higher than current levels among the US population.

Our study has multiple important strengths that include long follow-up, objective and repeated exposure ascertainment, and high quality of outcome and covariate data collected in the DPP and DPPOS. In addition, the successful execution of the lifestyle intervention in the DPP provides a unique opportunity to test our hypotheses.

## Conclusions

Among adults at high risk of type 2 diabetes, we observed that higher plasma PFAS concentrations were associated with a prospective and long-term increase in weight and hip girth among individuals randomized to a placebo group, but not for those randomized to a lifestyle intervention of diet and exercise. Exercise and a balanced diet confer many benefits; our results suggest that another benefit might be modification and attenuation of the obesogenic effects of environmental chemicals such as PFASs.

## Notes

Supplement. **eTable 1.** Geometric Means and Interquartile Ranges (IQR) of Plasma Perfluoroalkyl and Polyfluoroalkyl Substance (PFAS) Concentrations at Baseline, in Year 2, and Mean of Baseline and Year 2 Measured in the Diabetes Prevention Program (DPP) **eTable 2.** Sample Size, Mean Number of Years, and Range of Follow-up Since DPP Randomization for Weight Measurements From Baseline to the Second Phase of the Diabetes Prevention Program Outcomes Study (DPPOS) **eTable 3.** Estimated Adjusted Difference in Mean Weight Change From Baseline to the 5th Annual DPPOS Visit (Mean of 9-years of Follow-up Post Randomization), per Doubling in Baseline Plasma PFAS Concentrations and Stratified by Treatment Assignment **eTable 4.** Estimated Adjusted Difference in Mean Waist Circumference From Baseline to the 5th Annual DPPOS Visit (Mean of 9-Years of Follow-up Post Randomization), per Doubling in Mean Plasma PFAS Concentrations and Stratified by Treatment Assignment **eFigure 1.** A) Mean Change in Weight, B) Waist Circumference, and C) Hip Girth From Baseline Measurements at Each Study Visit by Baseline Treatment Assignment for Participants Included in the Current Study From the Diabetes Prevention Program (DPP) and Outcomes Study (DPPOS) **eFigure 2.** Spearman Correlation Coefficients (*r*_s_) for Mean Perfluoroalkyl and Polyfluoroalkyl Substances (PFASs) Plasma Concentrations Measured at Baseline and During the Second Year of the Diabetes Prevention Program (DPP) Among Individuals Included in this Study (N = 957) **eFigure 3.** A) Adjusted Estimated Change in Weight From Baseline at the 25th and 75th Percentiles of Total Baseline PFASs, B) Adjusted Estimated Change in Waist Circumference From Baseline at the 25th and 75th Percentiles of Total Baseline PFASs, and C) Adjusted Estimated Change in Hip Girth From Baseline at the 25th and 75th Percentiles of Total Baseline PFASs **eAppendix.** Longitudinal Regression Models
