# Supplementary material for: Association of Perfluoroalkyl and Polyfluoroalkyl Substances With Adiposity
Source: JAMA Netw Open. 2018 Aug 31;1(4):e181493. doi: 10.1001/jamanetworkopen.2018.1493 (PMC6324277; doi:10.1001/jamanetworkopen.2018.1493)
Supplement: Supplement. — eTable 1. Geometric Means and Interquartile Ranges (IQR) of Plasma Perfluoroalkyl and Polyfluoroalkyl Substance (PFAS) Concentrations at Baseline, in Year 2, and Mean of Baseline and Year 2 Measured in the Diabetes Prevention Program (DPP) eTable 2. Sample Size, Mean Number of Years, and Range of Follow-up Since DPP Randomization for Weight Measurements From Baseline to the Second Phase of the Diabetes Prevention Program Outcomes Study (DPPOS) eTable 3. Estimated Adjusted Difference in Mean Weight Change From Baseline to the 5th Annual DPPOS Visit (Mean of 9-years of Follow-up Post Randomization), per Doubling in Baseline Plasma PFAS Concentrations and Stratified by Treatment Assignment eTable 4. Estimated Adjusted Difference in Mean Waist Circumference From Baseline to the 5th Annual DPPOS Visit (Mean of 9-Years of Follow-up Post Randomization), per Doubling in Mean Plasma PFAS Concentrations and Stratified by Treatment Assignment eFigure 1. A) Mean Change in Weight, B) Waist Circumference, and C) Hip Girth From Baseline Measurements at Each Study Visit by Baseline Treatment Assignment for Participants Included in the Current Study From the Diabetes Prevention Program (DPP) and Outcomes Study (DPPOS) eFigure 2. Spearman Correlation Coefficients (rs) for Mean Perfluoroalkyl and Polyfluoroalkyl Substances (PFASs) Plasma Concentrations Measured at Baseline and During the Second Year of the Diabetes Prevention Program (DPP) Among Individuals Included in this Study (N = 957) eFigure 3. A) Adjusted Estimated Change in Weight From Baseline at the 25th and 75th Percentiles of Total Baseline PFASs, B) Adjusted Estimated Change in Waist Circumference From Baseline at the 25th and 75th Percentiles of Total Baseline PFASs, and C) Adjusted Estimated Change in Hip Girth From Baseline at the 25th and 75th Percentiles of Total Baseline PFASs eAppendix. Longitudinal Regression Models [file jamanetwopen-1-e181493-s001.pdf]

Supplementary Online Content

Cardenas A, Hauser R, Gold DR, et al. Association of perfluoroalkyl and polyfluoroalkyl substances with adiposity. *JAMA Netw Open*. 2018;1(4):e181493. doi:10.1001/jamanetworkopen.2018.1493

eTable 1. Geometric Means and Interquartile Ranges (IQR) of Plasma Perfluoroalkyl and Polyfluoroalkyl Substance (PFAS) Concentrations at Baseline, in Year 2, and Mean of Baseline and Year 2 Measured in the Diabetes Prevention Program (DPP)

eTable 2. Sample Size, Mean Number of Years, and Range of Follow-up Since DPP Randomization for Weight Measurements From Baseline to the Second Phase of the Diabetes Prevention Program Outcomes Study (DPPOS)

eTable 3. Estimated Adjusted Difference in Mean Weight Change From Baseline to the 5th Annual DPPOS Visit (Mean of 9-years of Follow-up Post Randomization), per Doubling in Baseline Plasma PFAS Concentrations and Stratified by Treatment Assignment

eTable 4. Estimated Adjusted Difference in Mean Waist Circumference From Baseline to the 5th Annual DPPOS Visit (Mean of 9-Years of Follow-up Post Randomization), per Doubling in Mean Plasma PFAS Concentrations and Stratified by Treatment Assignment

eFigure 1. A) Mean Change in Weight, B) Waist Circumference, and C) Hip Girth From Baseline Measurements at Each Study Visit by Baseline Treatment Assignment for Participants Included in the Current Study From the Diabetes Prevention Program (DPP) and Outcomes Study (DPPOS)

eFigure 2. Spearman Correlation Coefficients (r_s) for Mean Perfluoroalkyl and Polyfluoroalkyl Substances (PFASs) Plasma Concentrations Measured at Baseline and During the Second Year of the Diabetes Prevention Program (DPP) Among Individuals Included in this Study (N=957)

eFigure 3. A) Adjusted Estimated Change in Weight From Baseline at the 25th and 75th Percentiles of Total Baseline PFASs, B) Adjusted Estimated Change in Waist Circumference From Baseline at the 25th and 75th Percentiles of Total Baseline PFASs, and C) Adjusted Estimated Change in Hip Girth From Baseline at the 25th and 75th Percentiles of Total Baseline PFASs

eAppendix. Longitudinal Regression Models

This supplementary material has been provided by the authors to give readers additional information about their work.

© 2018 Cardenas A et al. *JAMA Network Open.*

eTable 1. Geometric Means and Interquartile Ranges (IQR) of Plasma Perfluoroalkyl and Polyfluoroalkyl Substance (PFAS) Concentrations at Baseline, in Year 2, and Mean of Baseline and Year 2 Measured in the Diabetes Prevention Program (DPP)

			**Baseline**			**Year 2**			**Mean PFASs**
**PFASs Analyte**	**Chemical Name**		**<LOD [a] n (%)**	**Geometric Mean (IQR) (ng/mL)**		**<LOD [a] n (%)**	**Geometric Mean (IQR) (ng/mL)**		**Geometric Mean (IQR) (ng/mL)**
**PFOS**	Perfluorooctane sulfonic acid		0	26.38 (22.80)		0	28.13 (19.2)		27.94 (19.45)
**PFOA**	Perfluorooctanoic acid		0	4.82 (3.20)		0	5.77 (4.0)		5.42 (3.50)
**PFHxS**	Perfluorohexane sulfonic acid		1 (0.1%)	2.41 (2.40)		9 (0.9%)	2.42 (2.4)		2.49 (2.25)
**Et-PFOSA-AcOH**	*N*-ethyl-perfluorooctane sulfonamido acetic acid		32 (3.3%)	1.13 (1.50)		76 (7.9%)	0.94 (1.3)		1.18 (1.35)
**Me-PFOSA-AcOH**	*N*-methyl-perfluorooctane sulfonamido acetic acid		29 (2.6%)	0.94 (1.10)		32 (3.3%)	1.03 (1.0)		1.09 (1.00)
**PFNA**	Perfluorononanoic acid		65 (6.8%)	0.53 (0.40)		46 (4.8%)	0.58 (0.5)		0.57 (0.45)
**Total sum PFASs**	Sum PFASs		---	38.44 (28.60)		---	41.12 (27.1)		40.57 (22.65)

LOD = Limit of Detection; LOD was 0.1 ng/mL for all analytes; IQR = Inter-Quartile Range

PFOS = n-PFOS + Sm-PFOS + Sm2-PFOS

PFOA = n-PFOA + Sb-PFOA

© 2018 Cardenas A et al. *JAMA Network Open.*

eTable 2. Sample Size, Mean Number of Years, and Range of Follow-up Since DPP Randomization for Weight Measurements From Baseline to the Second Phase of the Diabetes Prevention Program Outcomes Study (DPPOS)

*DPP/DPPOS VISIT	Mean time since DPP randomization (years)	Sample size	Range of years since DPP randomization	
BAS	0.00	957	0.00	0.00
M06	0.50	935	0.42	0.77
Y01	1.01	948	0.90	1.61
M18	1.50	933	1.39	1.77
Y02	2.01	954	1.75	2.37
M30	2.50	933	2.39	2.89
Y03	3.02	932	2.88	3.69
M42	3.51	849	3.41	3.94
Y04	4.02	608	3.86	4.52
M54	4.51	398	4.38	4.86
Y05	5.02	222	4.92	5.37
M66	5.50	62	5.42	5.56
01A	4.98	849	3.46	6.58
01M	4.99	811	3.42	6.78
02A	5.98	819	4.54	7.23
02M	5.99	800	4.46	7.63
03A	6.97	801	5.73	8.29
03M	6.98	782	5.36	8.73
04A	7.97	794	6.88	9.42
04M	7.98	770	6.45	9.74
05M	8.96	753	7.46	10.53
05A	8.98	802	7.89	10.68
06A	9.97	768	8.91	11.65
06M	9.98	751	8.45	11.80
07A	10.98	743	9.94	12.37
07M	11.00	732	9.46	12.65
08A	11.97	738	10.91	13.46
08M	11.98	729	10.46	13.58
09A	12.96	726	11.79	14.35
09M	12.98	720	11.46	14.56
10A	13.96	717	12.95	15.30
10M	13.97	709	12.46	15.63
11A	14.98	705	13.92	16.22
11M	14.98	688	13.42	16.56

*Labels used for annual and semiannual visits in DPP/DPPOS. BAS= Baseline, Y##= yearly visits during DPP, M##= mid-annual visits during DPP, ##A= annual visits during DPPOS and ##M= mid-year visits during DPPOS. DPPOS data includes a 13-month bridge period between DPP and DPPOS

© 2018 Cardenas A et al. *JAMA Network Open.*

eTable 3. Estimated Adjusted Difference in Mean Weight Change From Baseline to the 5th Annual DPPOS Visit (Mean of 9-years of Follow-up Post Randomization), per Doubling in Baseline Plasma PFAS Concentrations and Stratified by Treatment Assignment[a]

		Lifestyle Intervention Group (n=402)			Placebo Group (n=400)	
Baseline PFASs		Weight Change From Baseline per Doubling in PFASs (95% CI), Kg	*P* Value		Weight Change From Baseline per Doubling in PFASs (95% CI), Kg	*P* Value
Total baseline PFAS		-0.51 (-1.59, 0.57)	0.35		**1.21 (0.02, 2.41)**	**0.04**
PFOS		-0.52 (-1.50, 0.45)	0.29		**1.15 (0.07, 2.23)**	**0.03**
PFOA		-0.16 (-1.25, 0.92)	0.77		0.61 (-0.62, 1.86)	0.33
PFHxS		0.13 (-0.64, 0.89)	0.75		0.04 (-0.90, 0.98)	0.92
Et-PFOSA-AcOH		-0.09 (-0.71, 0.53)	0.77		0.41 (-0.27, 1.11)	0.24
Me-PFOSA-AcOH		0.12 (-0.62, 0.86)	0.76		0.31 (-0.51, 1.14)	0.46
PFNA		-0.04 (-0.86, 0.77)	0.91		**1.05 (0.14, 1.97)**	**0.02**

[a] Adjusted for participant sex, race/ethnicity, height, age (categorical), marital status (categorical), education (categorical), income (categorical) and smoking history (categorical)

© 2018 Cardenas A et al. *JAMA Network Open.*

eTable 4. Estimated Adjusted Difference in Mean Waist Circumference From Baseline to the 5th Annual DPPOS Visit (Mean of 9-Years of Follow-up Post Randomization), per Doubling in Mean Plasma PFAS Concentrations and Stratified by Treatment Assignment[a]

		Lifestyle *N*=402			Placebo *N*=398	
[b] Mean PFASs		cm per doubling in PFASs (95% CI)	*P* Value		cm per doubling in PFASs (95% CI)	*P* Value
Total mean PFASs		-0.39 (-1.54, 0.76)	0.50		0.34 (-0.99, 1.68)	0.61
PFOS		-0.46 (-1.53, 0.61)	0.39		0.46 (-0.77, 1.69)	0.47
PFOA		0.01 (-1.09, 1.10)	0.99		-0.52 (-1.80, 0.77)	0.43
PFHxS		-0.02 (-0.79, 0.75)	0.97		-0.41 (-1.38, 0.56)	0.41
Et-PFOSA-AcOH		-0.26 (-0.89, 0.37)	0.41		0.37 (-0.42, 1.15)	0.36
Me-PFOSA-AcOH		-0.13 (-0.97, 0.71)	0.75		0.43 (-0.60, 1.46)	0.41
PFNA		0.09 (-0.81, 1.00)	0.84		-0.04 (-1.06, 0.98)	0.94

[b] Mean of baseline and year-2 concentrations

[a] Adjusted for participant sex, race/ethnicity, height, age (categorical), marital status (categorical), education (categorical), income (categorical) and smoking history (categorical)

© 2018 Cardenas A et al. *JAMA Network Open.*

eFigure 1. A) Mean Change in Weight, **B**) Waist Circumference, and **C**) Hip Girth From Baseline Measurements at Each Study Visit by Baseline Treatment Assignment for Participants Included in the Current Study From the Diabetes Prevention Program (DPP) and Outcomes Study (DPPOS)

A)

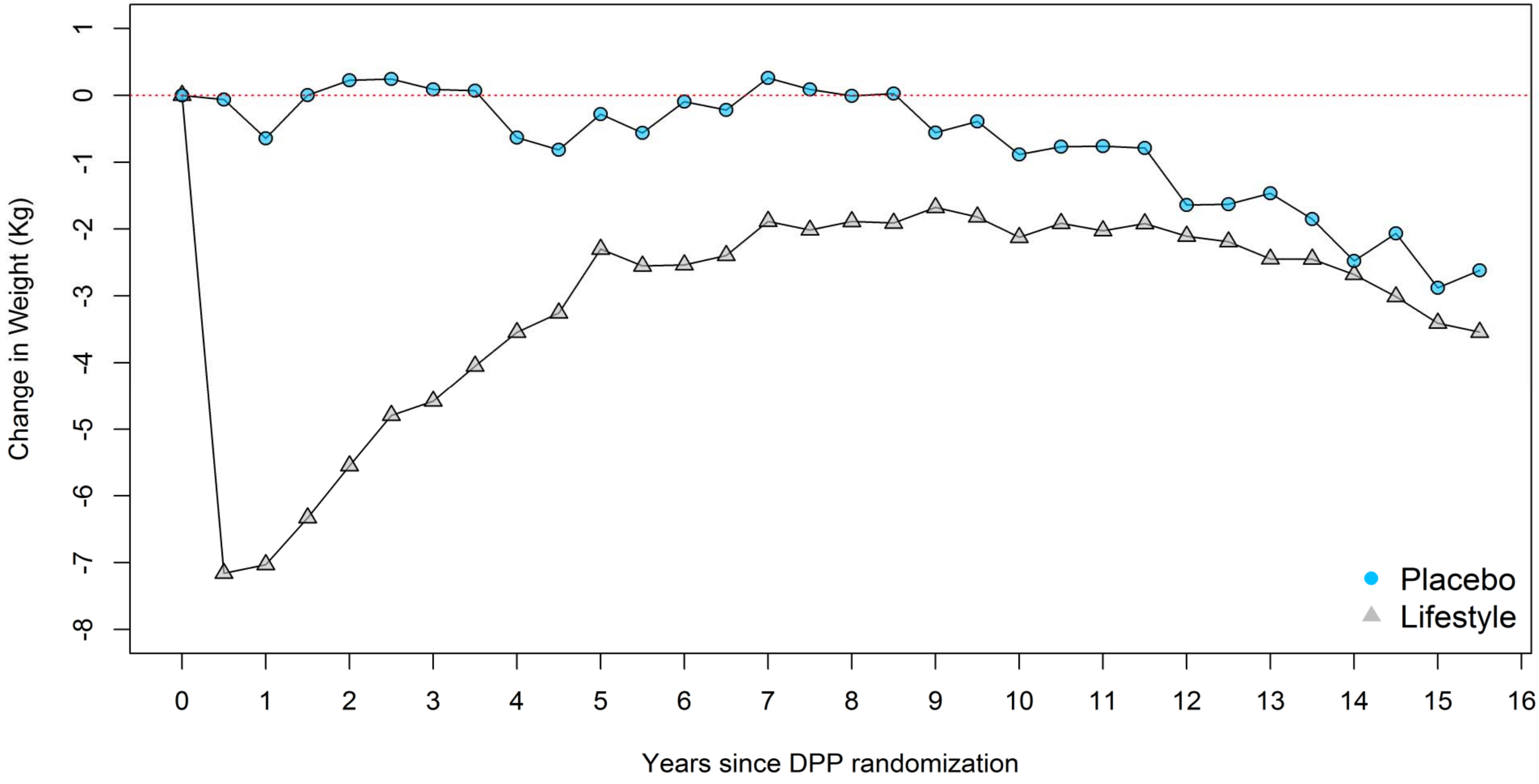

© 2018 Cardenas A et al. *JAMA Network Open.*

B)

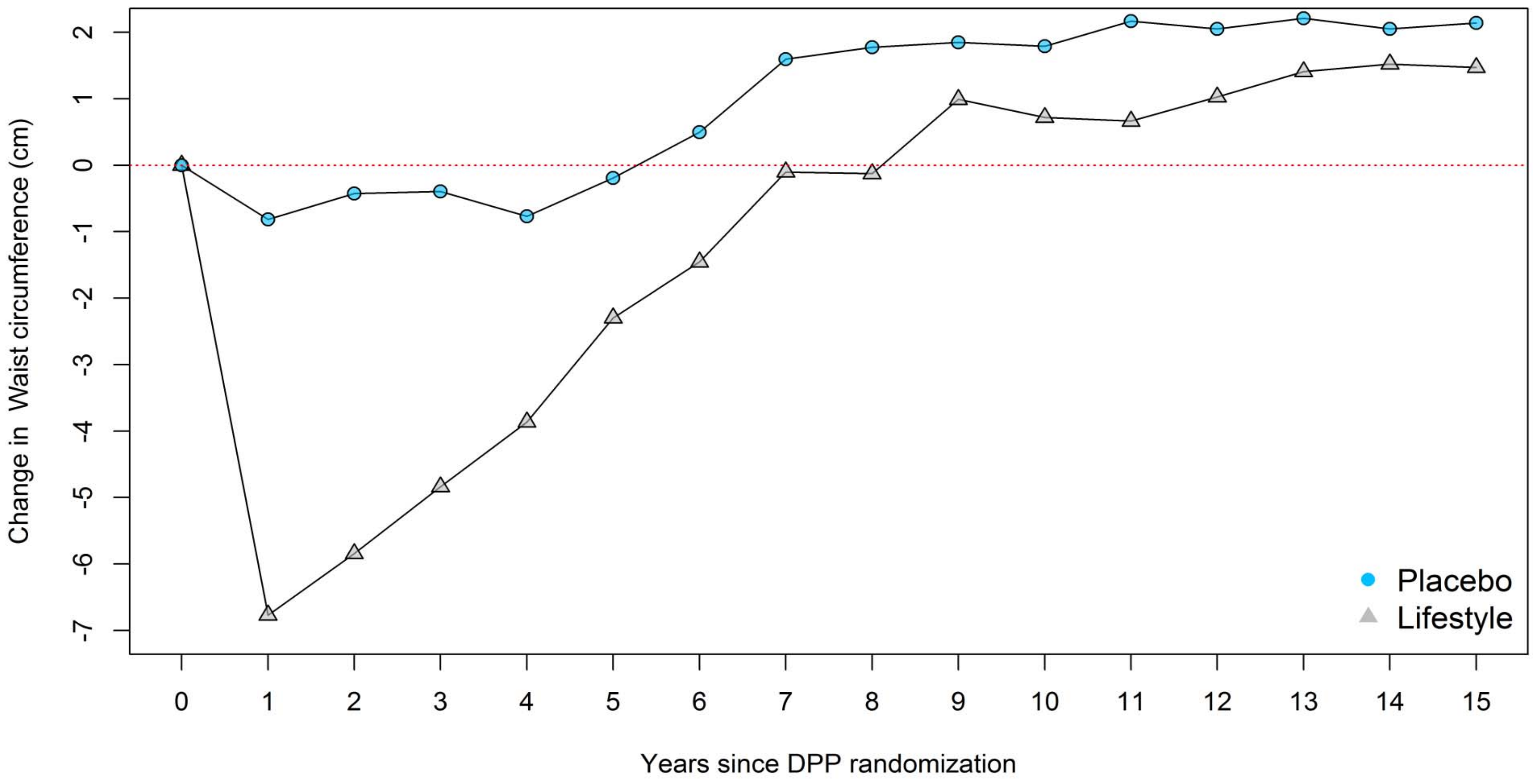

© 2018 Cardenas A et al. *JAMA Network Open.*

C)

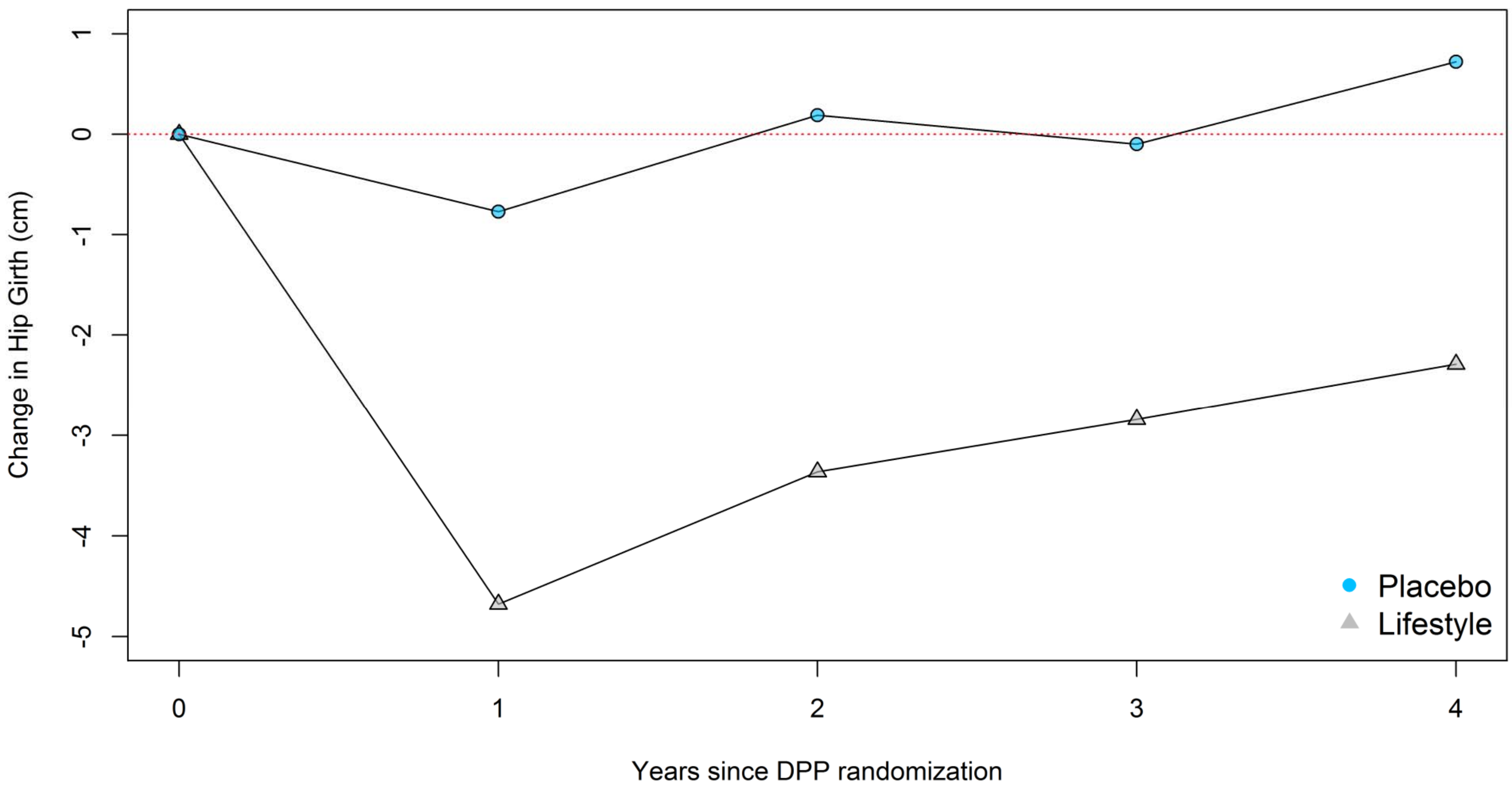

© 2018 Cardenas A et al. *JAMA Network Open.*

eFigure 2. Spearman Correlation Coefficients (r_s) for Mean Perfluoroalkyl and Polyfluoroalkyl Substances (PFASs) Plasma Concentrations Measured at Baseline and During the Second Year of the Diabetes Prevention Program (DPP) Among Individuals Included in this Study (N=957)

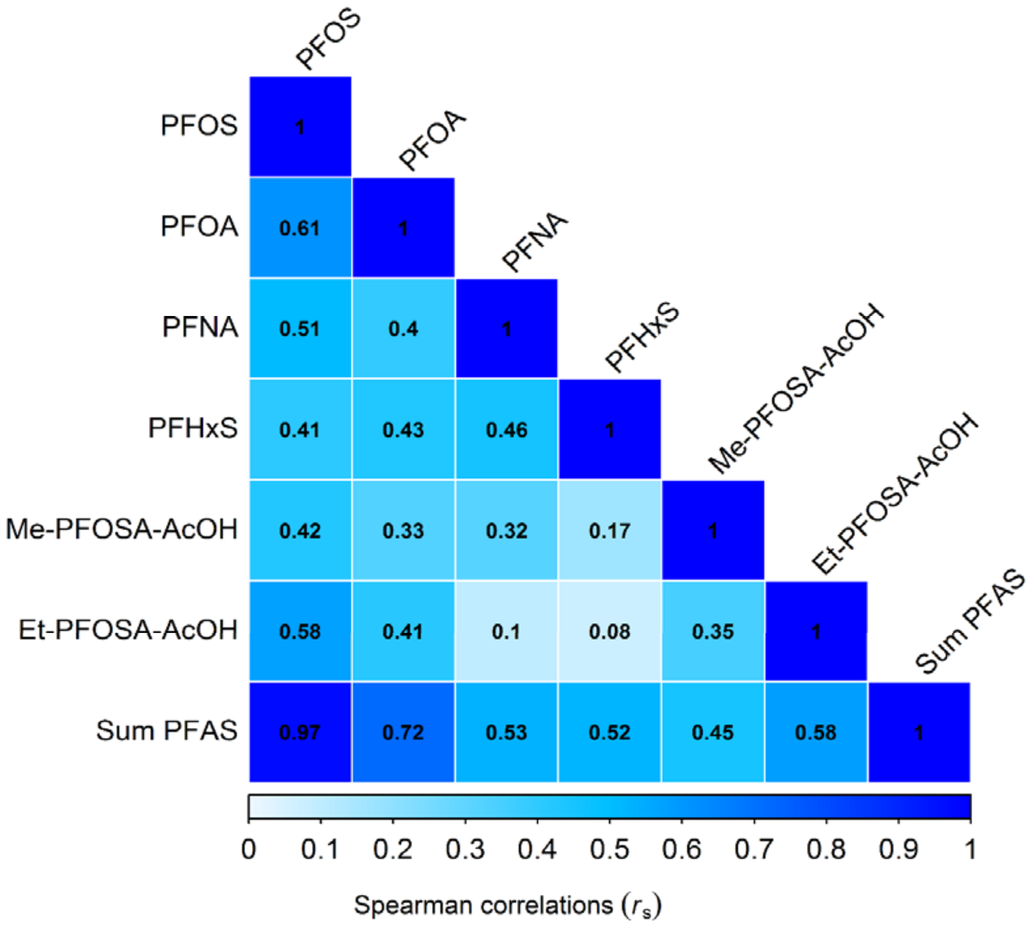

© 2018 Cardenas A et al. *JAMA Network Open.*

eFigure 3. A) Adjusted Estimated Change in Weight From Baseline at the 25th and 75th Percentiles of Total Baseline PFASs, **B)** Adjusted Estimated Change in Waist Circumference From Baseline at the 25th and 75th Percentiles of Total Baseline PFASs, and **C**) Adjusted Estimated Change in Hip Girth From Baseline at the 25th and 75th Percentiles of Total Baseline PFASs

A)

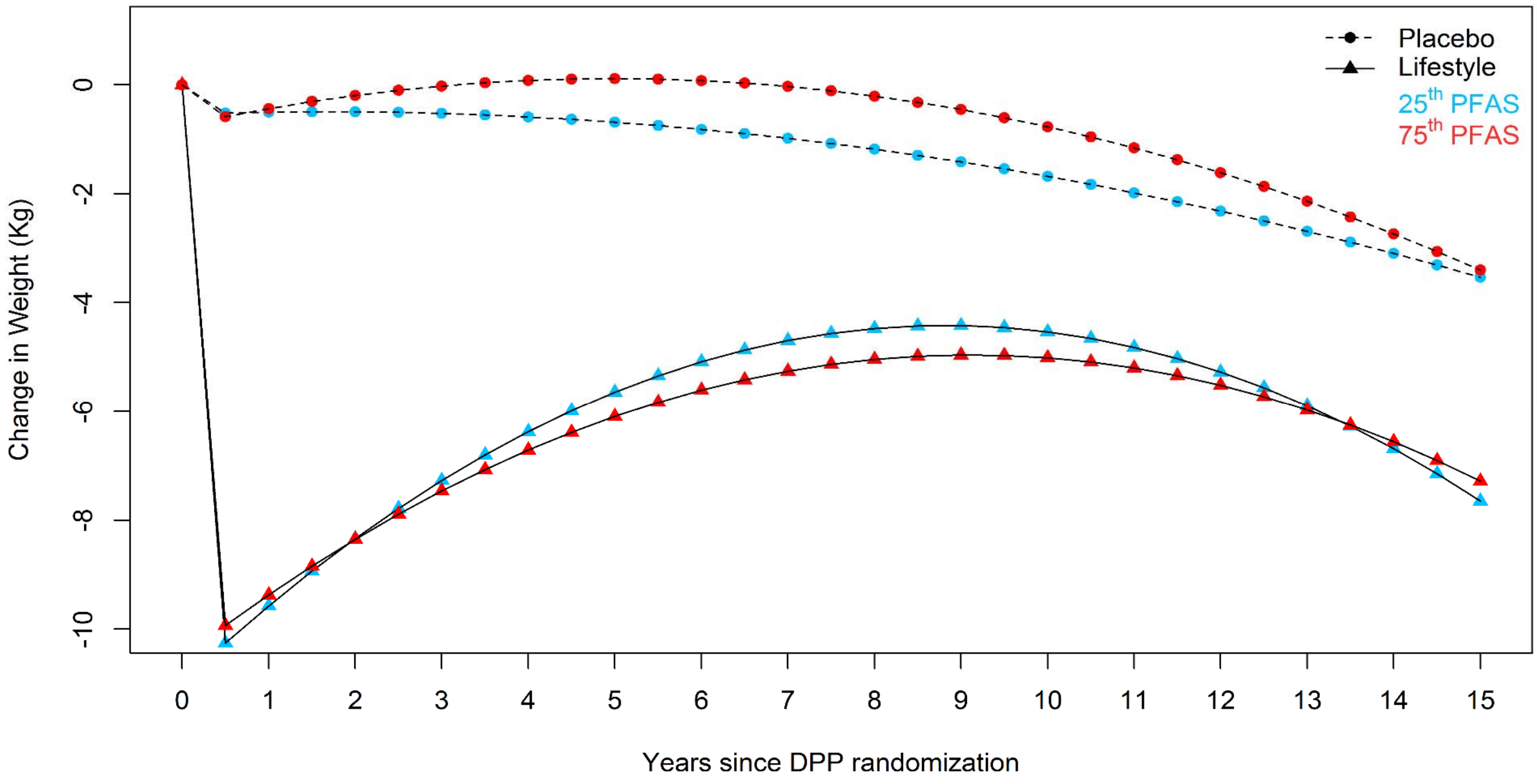

© 2018 Cardenas A et al. *JAMA Network Open.*

B)

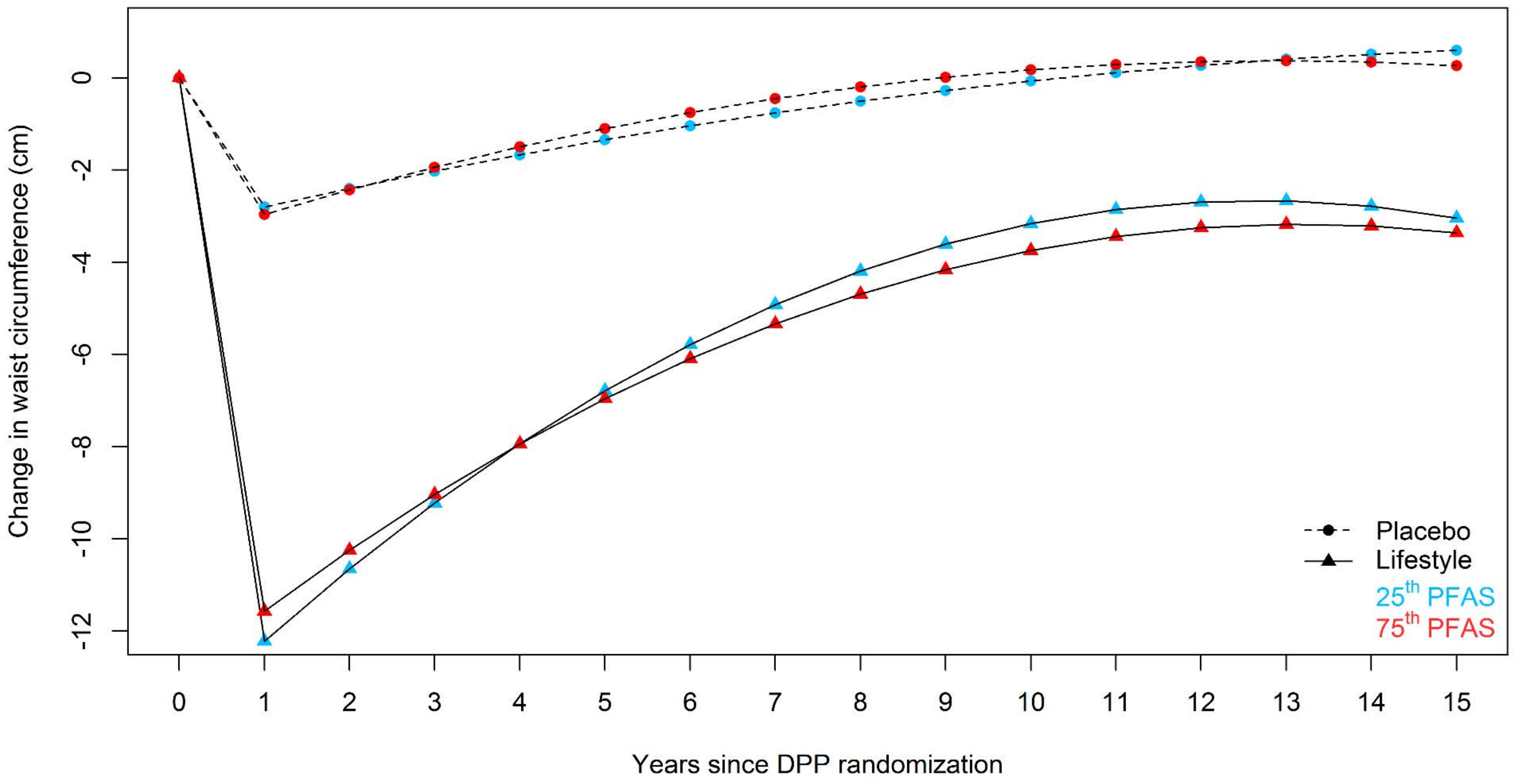

© 2018 Cardenas A et al. *JAMA Network Open.*

C)

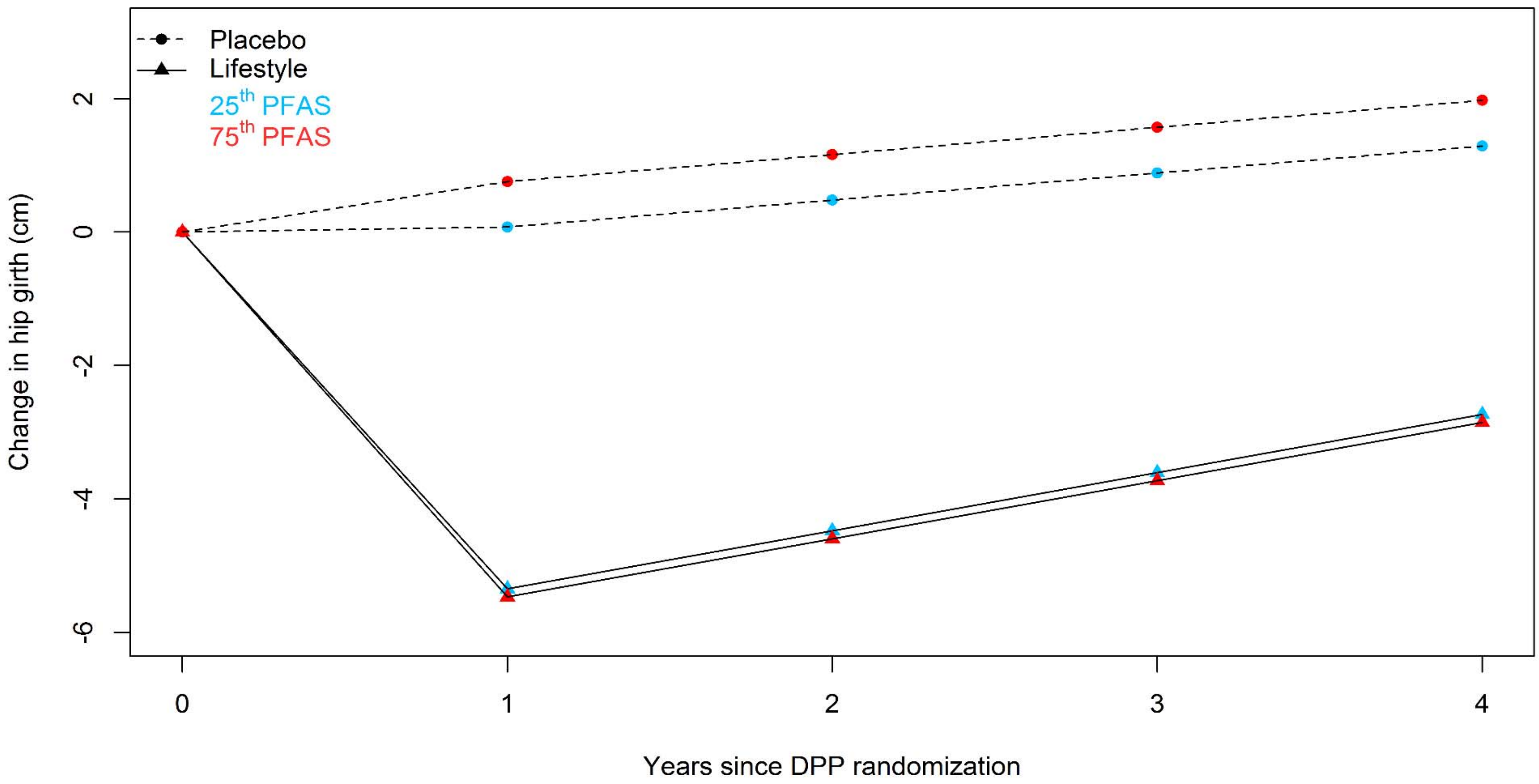

© 2018 Cardenas A et al. *JAMA Network Open.*

eAppendix. Longitudinal Regression Models

Testing for Statistical Interaction between PFASs and Treatment Assignment.

We tested for statistical interaction between the effect of $\log_2$-transformed PFAS over time and treatment assignment (Tx) in longitudinal models using three-way multiplicative interactions among PFAS, treatment group, time and time squared:

$$y_{ij} = \beta_0 + \beta_1 t_{ij} + \beta_2 t_{ij}^2 + \beta_3 \mathrm{PFAS}_i + \beta_4 \mathrm{Tx}_i +$$
$$\beta_5 t_{ij} * \mathrm{Tx}_i + \beta_6 t_{ij}^2 * \mathrm{Tx}_i + \beta_7 \mathrm{PFAS}_i * t_{ij} + \beta_8 \mathrm{PFAS}_i * t_{ij}^2 + \beta_9 \mathrm{PFAS}_i * \mathrm{Tx}_i +$$
$$\beta_{10} \mathrm{PFAS}_i * \mathrm{Tx}_i * t_{ij} + \boldsymbol{\beta_{11} \mathrm{PFAS}_i * \mathrm{Tx}_i * t_{ij}^2}$$
$$\ldots + b_{0i} + b_{1i} t_{ij} + e_{ij}$$

where i indexes the study participants and j the study visit number, y_{ij} is the change in weight or waist circumference from baseline for person i at visit number j, t_{ij} is the time since randomization in years at visit ij, PFAS_i is the baseline concentration for subject i, and Tx_i indicates treatment arm assignment (1 for lifestyle, 0 for placebo). The ellipsis indicates adjustment covariates. The model definition is completed by the assumption that e_{ij} is distributed normal and independent from (b_{0i}, b_{1i}), which is assumed multivariate normal.

Treatment Stratified Model: Change in Hip Girth from Baseline.

We generated effect estimates for $\log_2$-transformed PFAS, both mean and baseline concentrations, over the follow-up time in DPP for change in hip girth from baseline using the following model equation:

$$y_{ij} = \beta_0 + \beta_1 t_{ij} + \beta_2 \mathrm{PFAS}_i + \cdots + b_{0i} + b_{1i} t_{ij} + e_{ij}$$

The notation and assumptions from the first model equations are unchanged.

Treatment Stratified Model: Change in Weight and Waist Circumference from Baseline.

Significant, nonlinear, quadratic interactions between time and PFASs were observed for change in weight and waist circumference from baseline measurements over time, therefore we use the following model instead that allow PFASs to have non-linear effects over time:

$$y_{ij} = \beta_0 + \beta_1 t_{ij} + \beta_2 t_{ij}^2 + \beta_3 \mathrm{PFAS}_i + \beta_4 \mathrm{PFAS}_i * t_{ij} + \beta_5 \mathrm{PFAS}_i * t_{ij}^2 + \cdots + b_{0i} + b_{1i} t_{ij} + e_{ij}$$

The notation and assumptions from the first model equations are unchanged.

© 2018 Cardenas A et al. *JAMA Network Open.*
